# Cerebellar TMS Induces Motor Responses Mediating Modulation of Spinal Excitability: A Literature Review

**DOI:** 10.3390/brainsci13040531

**Published:** 2023-03-23

**Authors:** Akiyoshi Matsugi

**Affiliations:** Faculty of Rehabilitation, Shijonawate Gakuen University, Osaka 574-0011, Japan; a-matsugi@reha.shijonawate-gakuen.ac.jp; Tel.: +81-72-863-5043

**Keywords:** cerebellum, transcranial magnetic stimulation, electromyograph, spinal reflex, extrapyramidal tract, muscle tones

## Abstract

Since individuals with cerebellar lesions often exhibit hypotonia, the cerebellum may contribute to the regulation of muscle tone and spinal motoneuron pool excitability. Neurophysiological methods using transcranial magnetic stimulation (TMS) of the cerebellum have been recently proposed for testing the role of the cerebellum in spinal excitability. Under specific conditions, single-pulse TMS administered to the cerebellar hemisphere or vermis elicits a long-latency motor response in the upper or lower limb muscles and facilitates the H-reflex of the soleus muscle, indicating increased excitability of the spinal motoneuron pool. This literature review examined the methods and mechanisms by which cerebellar TMS modulates spinal excitability.

## 1. Introduction

**Figure 1:**
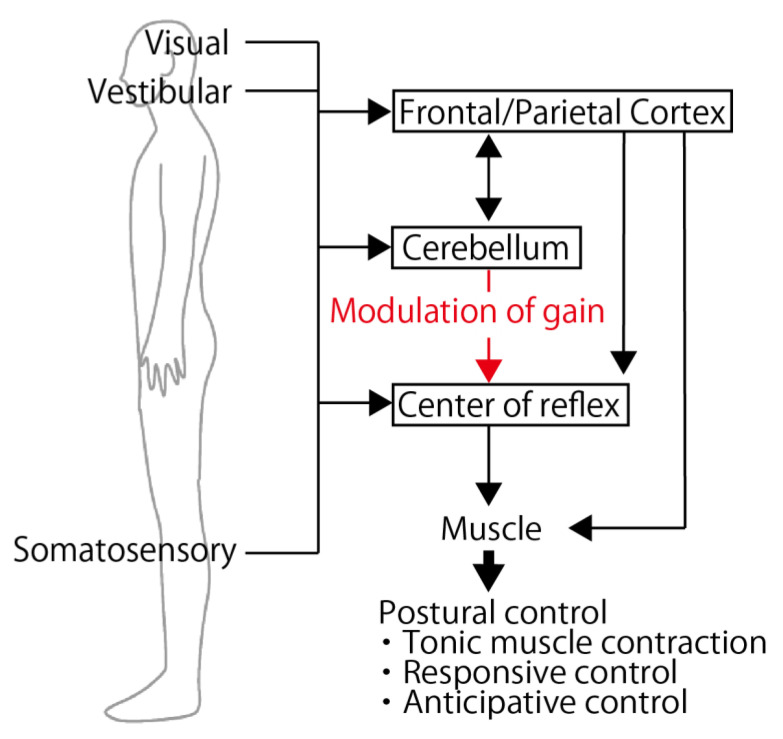
Brief schematic summary of the role of the cerebellum in postural control. The cerebellum modulates the gain in the spinal reflex and the excitability of the motoneuron pool. In particular, responsive postural control and muscle tones are under cerebellar control.

The cerebellum, in conjunction with the cerebral motor cortex and spinal cord, plays a crucial role in facilitating smooth motor execution. Damage to these neural circuits can result in ataxia, a condition characterized by impaired movement coordination [1]. Individuals with cerebellar infarction, for instance, often exhibit impaired postural control and gait as well as hypotonia of postural muscles [2]. Figure 1 illustrates the role of the cerebellum in postural control. Visual, vestibular, and somatosensory information triggers postural responses through reflex centers [3]. The spinal cord is also significantly involved in posture and gait, and the functional connection between the cerebellum and spinal cord is believed to be a key neuronal component of these processes. However, noninvasive in vivo methods for evaluating the functional connections between the cerebellum and spinal cord in humans have not yet been developed.

An evaluation of the functional connectivity between the cerebellum and nuclei in the brainstem and spinal motoneurons was previously performed using electrical stimulation in animals. In anesthetized cats, stimulation of the cerebellar deep nuclei induces action potentials in the vestibular nuclei and reticular formation [4]. In alert baboons, electrical stimulation of the cerebellar deep nuclei induces simple and synergistic motor responses in the limbs [5]. In Cebus monkeys, a non-somatotopic motor response is induced in the proximal limb by electrical stimulation of the dentate nucleus and interposed nuclei [6]. In humans, during posterior fossa surgery, electrical stimulation of the surface of the cerebellum is associated with motor responses in the facial and limb muscles on an electromyogram (EMG) [7]. However, these invasive methods have limitations and are not always feasible in vivo in human studies.

In this regard, noninvasive brain stimulation techniques such as transcranial magnetic stimulation (TMS) have been recently used to examine the functional connectivity between different sites in the brain, such as the cerebellum and the contralateral primary motor cortex [8]. In this literature review, the potential of using single-pulse TMS to test the effects of cerebellar stimulation on spinal excitability is discussed, and the results of previous studies using this method are reviewed.

## 2. Search Strategy

The literature search strategy utilized to investigate this subject was conducted through PubMed, using the terms “transcranial magnetic stimulation” and “cerebellum”. As of 24 December 2022, 572 articles were identified. The criteria for inclusion in this review article were the following: (1) human subjects only; (2) the outcome evaluated was a motor response on electromyography and/or the H-reflex. In the end, eleven articles were included (Table 1 and Table 2).

## 3. Cerebellar TMS

**Figure 2:**
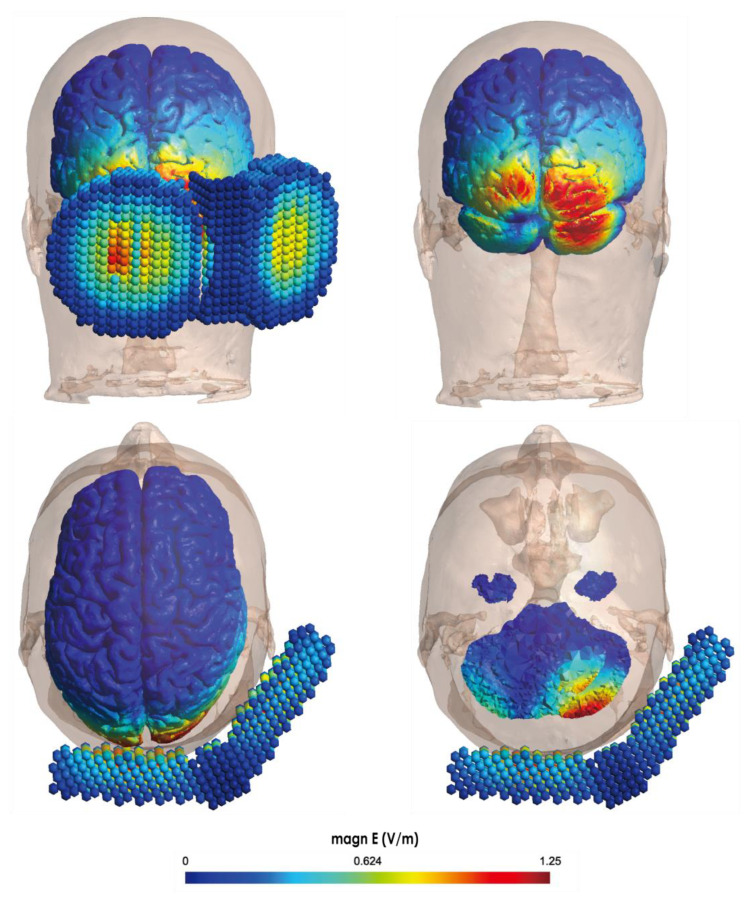
Visual simulation of the magnitude of the electrical fields on the right cerebellar hemisphere produced by a double-cone coil. The upper line shows images in the coronal view, and the bottom line shows images in the horizontal view. The two circular plates indicate the double-cone coil. magnE indicates the magnitude of the electrical field. The red area indicates the region with the strongest electrical field, and the blue area indicates the region of the lowest magnitude. The difference in values means that there is a difference in relative strength. These simulations display the magnitude of the electrical field that can be induced by TMS in the deep cerebellar hemispheres.

Transcranial Magnetic Stimulation (TMS) is a widely used technique for assessing neural circuit function within the brain [9,10]. It is used to investigate motor control in the primary motor cortex [11,12], test neuromodulation treatments [13,14], and probe cerebellar function for motor control by applying TMS over the posterior fossa [8,15]. Figure 2 presents a simulation of the cerebellar electrical field generated by the default settings of the SimNIBS software (ver. 4.0.0) [16] as well as the magnitude of the electrical field in the cerebellum. The D-B80 coil (with an inner diameter of 66 mm and an outer diameter of 95 mm, MagVenture Inc, USA) was used for the simulation. The cerebellum is situated deeper in the head than the primary motor cortex [17], and thus, a double-cone coil that could induce the magnetic field at a distance from the coil surface was employed to stimulate the cerebellar structure with accuracy [18]. This simulation suggests that TMS using a double-cone coil can provide some insights into an electrical field in the cerebellum: (1) One hemisphere can be stimulated selectively. (2) The electric field generated is relatively localized to the superficial cerebellar cortex, and it is difficult to generate a strong electric field near the brainstem, which is deeper. (3) It is possible to generate electric fields in the more superficial occipital lobes than in the cerebellar cortex, as previous studies have shown [17]. These advantages and concerns need to be understood before application.

The excitability of the corticospinal tract associated with the target muscle can be evaluated by measuring the amplitude of the motor-evoked potential (MEP) recorded on an EMG of the right first dorsal interosseous (FDI) muscle in response to single-pulse TMS applied over the left motor cortex. Additionally, inhibiting the MEPs evoked by left-M1-TMS by TMS of the right cerebellum (cerebellar brain inhibition: CBI) allows the evaluation of cerebellar cortex excitability and of the functional connection between the cerebellum and motor cortex [8,19,20]. CBI is absent in patients with damage to the cerebellum [21] or the dentate-thalamocortical pathway [22], indicating that CBI originates from the cerebellar structure and output pathway. Furthermore, CBI is influenced by motor learning [12], motor imagery [23], and neuromodulation montage [19,24]. Based on these findings, cerebellar TMS can be considered an effective approach for assessing cerebellar output.

The location of the coil is a crucial factor in obtaining the effect of cerebellar stimulation. The difference in distance from the scalp to the cerebellar gray matter is observed around the inion [17], and the effect of the site of the coil position on CBI is observed [8]. Figure 2 illustrates the electrical field in the brain produced by TMS with a double-cone coil on the right side of the inion. TMS of the site between the inion and the right mastoid process can induce CBI of the right FDI muscle, but CBI was absent at other sites [8]. On the other hand, the effect of disturbing adaptive motor learning concerning eye-head coordination movements can be obtained by TMS to the site under 1 cm from the inion [25]. The center of the cerebellum contributes to the control of eye movement [26]. Therefore, the position of the coils is crucial since the effect obtained depends on the stimulation position.

## 4. Short-Latency Motor Responses following TMS over the Posterior Fossa

High-intensity TMS, which is higher than the resting motor threshold for inducing cervicomedullary MEPs by TMS around the inion, can induce the motor response in hand muscles with a latency of approximately 20 ms [27]. However, these most likely originate from the corticospinal tract in the brainstem [28], because these short-latency MEPs are not typical of the cerebellum [15,28]. TMS of the posterior fossa induces an eddy current inside the cranium [29], and resultingly, this current can stimulate the brainstem on the foramen magnum level [28,30]. On the other hand, MEP does not appear after a single-pulse low-intensity TMS, which is of a threshold that is lower than the resting motor threshold, around the inion in a single-session paradigm [31]. However, MEP in an averaged and rectified EMG can be observed [15], indicating that the activation of descending and/or ascending pathways may confound the effect of TMS over the posterior fossa.

## 5. Long-Latency Motor Responses following Cerebellar TMS

Cerebellar TMS can modulate the excitability of the contralateral motor cortex, but cannot induce short-latency MEPs. However, some studies have reported a motor response in the proximal limb in animals by invasive direct electrical stimulation [5,6,7]. Some recent studies reported a long-latency motor response on an EMG by cerebellar TMS using a noninvasive method [31,32,33,34,35,36]. Interestingly, these motor responses appeared under specific conditions, which modulated their latency or probability of appearance (Table 1).

The first study that revealed this phenomenon was carried out by Sakihara et al. who reported that TMS around the inion induces a bilateral motor response on an EMG in the soleus muscles with a latency of approximately 100 ms [33], and that this latency was shortened by optokinetic stimulation [32]. Optokinetic stimulation drives the vestibulospinal response due to the illusion of falling. Therefore, this long-latency motor response on an EMG induced by cerebellar TMS of the soleus muscle may be mediated by extrapyramidal tracts such as the vestibulospinal tract. Subsequently, Hosokawa et al. reported that this long-latency motor response induced by cerebellar TMS was found in the extensor carpi radialis (ECR) muscle, and that its latency was modulated by the degree of postural control and drowsiness [36]. Their findings indicate that this long-latency motor response may be mediated by the brainstem’s reticular formation because the reticulospinal tract is activated during postural control [37], and the reticulothalamic pathway is deactivated depending on the degree of drowsiness [38]. Based on a series of reports by Yorifuji and colleagues [32,33,36], extrapyramidal tracts, especially the vestibulospinal and reticulospinal tracts, are thought to be involved in the long-latency motor response to cerebellar TMS. However, this response can be confirmed only with a very specific low-frequency (2–20 Hz) bandpass filter.

In another study by Hiraoka et al., a long-latency motor response was observed upon an EMG of the right FDI muscle during a visually guided tracking task with the right index finger, which required predictive eye–hand coordination [39], after right cerebellar TMS [34], but this response had a low probability of appearance in a non-tracking task [31,34]. Finger movement control based on visual feedback of the position of the finger and of the target was conducted using a cerebellar internal model [40]. Therefore, cerebellar activity may increase during this visually guided manual tracking task [39], and this task-dependent appearance of responses shows an association with cerebellar activity. This long-latency motor response of the FDI muscle was induced not only by stimulation of the right cerebellum but also by stimulation of the left and the center of the cerebellum [35], as reported by Hosokawa et al. [36]. A series of reports by Hiraoka and colleagues [31,34,35] suggested that cerebellar activity may be involved in the appearance of this long-latency motor response along with bilateral descending pathways, such as extrapyramidal tracts.

What these studies have in common is that they describe motor responses with long latencies that can be elicited by a wide range of cerebellar stimuli only during specific tasks such as standing or visually guided tracking. The extremely long latencies of approximately 100 ms for the lower limb and 60–90 ms for the upper limb indicate that these responses occur through a disynaptic neural circuit. The fact that these responses are observed in contralateral muscles, even with contralateral cerebellar stimulation, suggests that bilateral descending tracts may be involved. Typical bilateral tracts include the vestibulospinal and reticulospinal tracts, which have reciprocal projections to the left and right sides and are possible representative extrapyramidal tracts.

Because these motor responses are stochastic in appearance, 20–30 trials are required to elicit these responses, which have to be confirmed with an additive mean waveform or by checking the rate of appearance. They also tend to appear specifically during tasks related to cerebellar or extrapyramidal activity, making them difficult to confirm in a resting state, such as during surgery.

**Table 1:** Studies of cerebellar TMS and motor responses.

Author	Year	Coil	Target Muscle	Outcome	Latency	Findings
Sakihara et al. [33]	2003	F8	Soleus	Motor response	about 100 ms	Dependency of the stimulation site
Sakihara et al. [32]	2007	DC	Soleus	Motor response	about 100 ms	Modulation by optokinetic stimulation
Hiraoka et al. [34]	2010	DC	Rt-FDI	Motor response	about 90 ms	Task dependency
Matsugi et al. [35]	2012	DC	Rt-FDI	Motor response	about 90 ms	No dependency of the stimulation site in the cerebellum
Matsugi et al. [31]	2013	DC	Rt-FDI	Motor response	about 80 ms	Appearance depended on the task
Hosokawa et al. [36]	2014	DC	Bilateral ECR	Motor response	Ipsilateral 60 ms, contralateral 70 ms	Affected by postural control, drowsiness

Note: F8, figure-eight coil; DC, double cone coil; FDI, first dorsal interosseous; ECR, extensor carpi radialis; Rt, right.

## 6. Modulation of Spinal Excitability after Cerebellar TMS

The cerebellum shows anatomical [41] and functional [4] connectivity to spinal motoneurons via the brainstem nuclei. Thus, the hypothesis that cerebellar TMS modulates the excitability of the spinal motoneuron pool even in the resting state [7], since it was able to elicit motor responses during surgery, is quite tenable.

Regarding the method of testing the functional connectivity between the cerebellum and other brain sites, CBI is the most used [20,42]. Motor cortex excitability is estimated using MEP by single-pulse TMS over the primary motor cortex, and this potential is suppressed by prior conditioning with TMS over the contralateral cerebellum [8]. This conditioning-test stimulation paradigm has been used to probe functional connectivity [43]. Testing spinal excitability is required to assay cerebellar–spinal connectivity. Spinal motoneuron pool excitability can be measured using the H-reflex [44].

**Figure 3:**
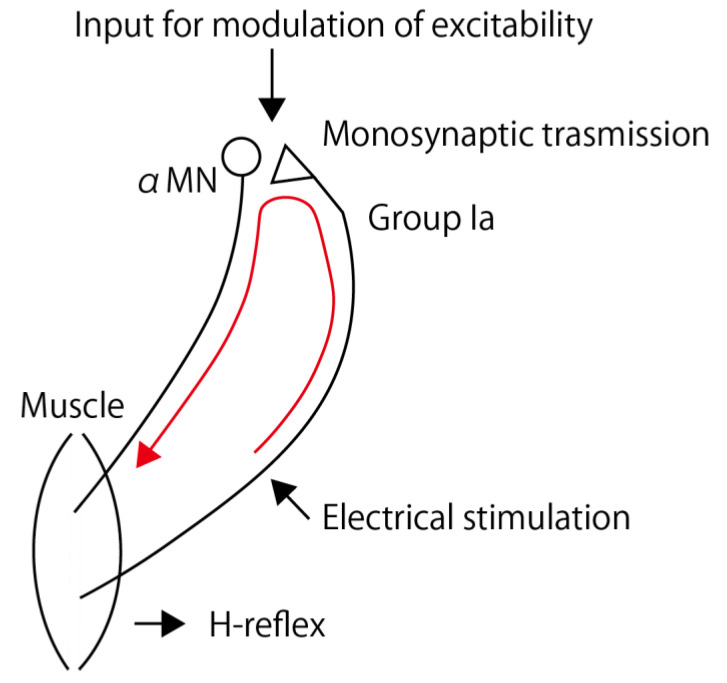
Circuit of the H-reflex. Electrical stimulation elicits an action potential in the group Ia afferent fibers of the sensory nerve. These ascending potentials activate the alpha motoneurons (αMN) in the spinal anterior horn through a monosynaptic pathway, ultimately inducing a single muscle twitch. This particular response is commonly referred to as the Hoffman reflex (H-reflex). The modulation of the H-reflex’s excitability is achieved through inputs delivered to the motoneuron pool via both pyramidal and extrapyramidal descending tracts.

The H-reflex is a monosynaptic spinal reflex that reflects the excitability of the spinal motoneuron pool (Figure 3) [45]. The soleus muscle is usually selected as the target muscle for the observation of the H-reflex [44]. The electrical stimulation of the tibial nerve induces the ascending action potential in group Ia fibers of the peripheral sensory nerve. This ascending potential activates the motoneurons by monosynaptic transmission. Some input is delivered to achieve the modulation of the excitability of the motoneuron pool via pyramidal and extrapyramidal descending tract [44]. Therefore, it is considered that the change in amplitude of the H-reflex reflects the change in the excitability of the spinal motoneuron pool’s excitability [45].

**Figure 4:**
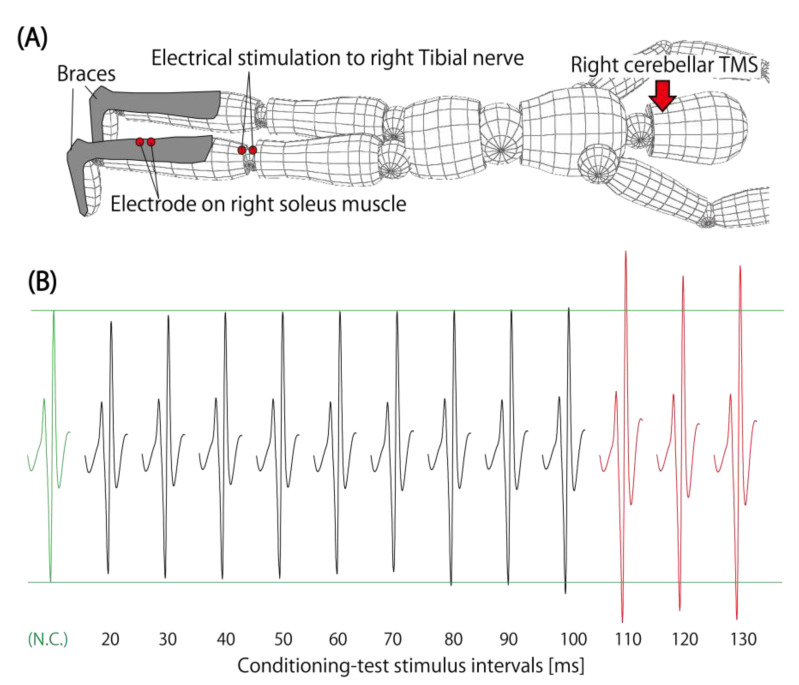
Typical setting used to test CSpF (**A**) and typical waveform of H-reflex conditioned by cerebellar TMS (**B**). In **A**, a single-pulse TMS is administered to the right cerebellar hemisphere prior to the application of electrical stimulation to the right tibial nerve in order to elicit the H-reflex on the right soleus muscle. The location of TMS is indicated by a red arrow, while red circles illustrate the location of the electrode used for electrical stimulation and EMG recording. In **B**, the H-reflex waveforms in each conditioning-test stimulus interval are described. The green waveform denotes the non-conditioned (N.C.) H-reflex, while the other waveforms represent the conditioned H-reflex, which are normalized by N.C. H-reflex amplitude. The green horizontal lines indicate the amplitude of the non-conditioned H-reflex (control condition), and the distance between green lines indicate 100% of N.C. H-reflex amplitude. The red waveforms at the 110–130 ms intervals signify the H-reflex, which is significantly facilitated by cerebellar TMS, as reported in a previous study [46]. It is worth noting that these settings and waveforms were initially generated based on the aforementioned study [46] for this review manuscript and were not previously published.

Figure 4 indicates the typical setting used to test this cerebellar spinal facilitation (CSpF) and the typical waveform of the H-reflex of the soleus muscle. Some previous studies have reported that the H-reflex is facilitated by cerebellar TMS (Table 2). One study reported that the right soleus’ H-reflex was facilitated by conditioning TMS over the cerebellum [46]. The interval between conditioning and the test stimulation, which involved electrical tibial nerve stimulation to induce the H-reflex, was 110–130 ms to observe the significant facilitation of the H-reflex [46]. The amount of CSpF was modulated by an externally paced finger-tapping task, which requires cerebellar contribution in the time management of motor excursion according to external cues [2]. Furthermore, cerebellar transcranial direct current stimulation (tDCS) modulates the amount of facilitation in a polarity-dependent manner [47]. These findings indicate that the facilitation of spinal excitability after cerebellar TMS may be affected by the activity of the cerebellum. The possible pathway to the spinal motoneuron pool from the cerebellum may involve extrapyramidal tract projections to the interneurons associated with the presynaptic inhibition (PSI) of group Iα, but not with reciprocal inhibition [48]. Furthermore, TMS to the contralateral cerebellar hemisphere and the center of the cerebellum can induce this facilitation of the excitability of the spinal motoneuron pool [49]. These findings indicate that the possible descending pathways involved are the vestibulospinal, reticulospinal, and rubrospinal tracts because the cerebellum shows anatomical projections to the spinal motoneuron pool [50] via vestibular nuclei [51], red nuclei [52], and the reticular formation [53] of the brainstem. Interestingly, this CSpF was also observed in patients with spinocerebellar degeneration (SCD) without CBI [21], indicating that the dentate-thalamocortical pathway may not be needed for CSpF.

**Table 2:** Studies of single pulse cerebellar TMS and spinal reflex.

Author	Year	Coil	Target Muscle	Outcome	Latency	Findings
Matsugi et al. [46]	2014	DC	Rt-Soleus	Modulation of H-reflex	ISI 110–130 ms	Time course of ISI, task dependency
Matsugi et al. [48]	2015	DC	Rt-Soleus	Modulation of H-reflex	ISI 110 ms	Mediation of PSI, not of RI
Matsugi [49]	2018	DC	Rt-Soleus	Modulation of H-reflex	ISI 110 ms	No dependency of stimulation site in the cerebellum
Matsugi et al. [21]	2018	DC	Rt-Soleus	Modulation of H-reflex	ISI 110 ms	Facilitation in SCD with CBI-absent
Matsugi et al. [47]	2020	DC	Rt-Soleus	Modulation of H-reflex	ISI 110 ms	Dependency of tDCS-polarity

Note: DC, double cone coil; Rt, right; ISI, inter-stimulus interval; PSI, presynaptic inhibition; RI, reciprocal inhibition; SCD, spinocerebellar degeneration; CBI, cerebellar brain inhibition; tDCS, transcranial direct current stimulation.

## 7. Possible Pathway

**Figure 5:**
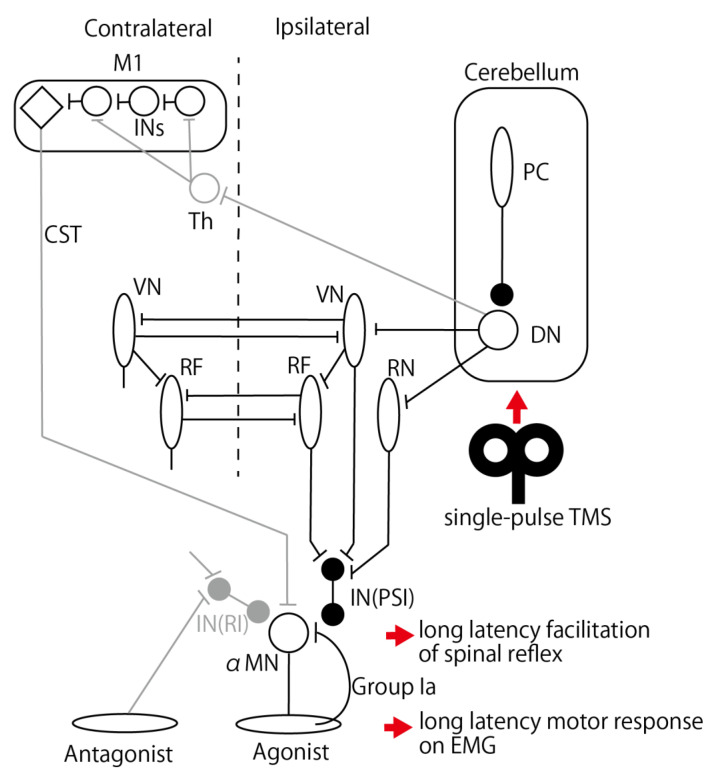
Possible pathway underlying the long-latency motor response on an EMG and the long-latency facilitation of the spinal reflex following cerebellar conditioning TMS. The black line indicates the possible pathway, and the gray line indicates the redundant pathways. M1, primary motor cortex; Th, thalamus; CST, corticospinal tract; PC, Purkinje cell; DN, deep cerebellar nucleus; VN, vestibular nuclei; RF, reticular formation; RN, red nuclei; IN, interneuron; PSI, presynaptic inhibition; RI, reciprocal inhibition; α-MN, alpha motoneuron; Agonist, agonist’s muscle; Antagonist, antagonist’s muscle; TMS, transcranial magnetic stimulation.

The findings described above indicate a possible pathway associated with motor responses and CSpF. Consistently, these responses are bilateral and have similar long-latency responses, and because the projection pathway to the contralateral motor cortex does not need to be intact, the pathway shown in Figure 5 is proposed.

This long-latency motor response of about 100 ms and characterized by the facilitation of the excitability of spinal reflex is unlikely to involve the corticospinal tracts. If the corticospinal tracts in the brainstem were to be directly stimulated by TMS over the occipital region, a short latency motor response, similar to the cervico-medullary MEP with a latency of about 20 ms, would be induced [15]. Therefore, the corticospinal tract may not be involved in these longer-latency responses. The next important tract that could be responsible for these phenomena, the contralateral motor cortex-mediated pathway, is also unlikely to be involved. The long-latency facilitation of the H-reflex was achieved even in SCD patients with absent CBI [21], indicating that the dentate-thalamocortical pathway [22] may not be needed to obtain the effects of cerebellar TMS to facilitate the excitability of the spinal reflex, because CBI absence indicates the dysfunctional condition of the dentate-thalamocortical pathway [8]. The third possible path, the one associated with reciprocal inhibition of spinal interneurons may also not be involved. This is based on the evidence that reciprocal inhibition is not affected by cerebellar TMS [48]. Based on these findings, the corticospinal pathway, dentate-thalamocortical pathway and interneurons associated with reciprocal inhibition may be excluded for long-latency motor responses mediating the modulation of spinal motoneuron pool excitability.

Since the response is not mediated by the corticospinal tracts, these responses and the elicitation of the H-reflex most likely originate in the extrapyramidal tracts. The descending spinal tracts with projections to interneurons involved in PSI are the vestibulospinal tract, reticulospinal tract, and rubrospinal tract. The left and right vestibulospinal nuclei and reticular formation in the brainstem are monosynaptically coupled and can easily propagate action potentials to contralateral nuclei or formations. Some previous studies reported the motor response that was induced by TMS over the ipsilateral and contralateral site of the target muscle [32,33,35,36], and the induction of CSpF [49]. Therefore, these bilateral extrapyramidal tracts may have been involved in the production of the motor response and the CSpF observed in the previously cited studies.

The very long latency suggests that this response depends on transmission through several neural circuits. In other words, the effect may be exerted by projections from the vestibular nucleus, reticular formation, and red nucleus, which receive direct input from the cerebellum, to interneurons involved in Iα PSI via multiple synapses. Further studies are needed to determine whether additional neural circuits are at play.

## 8. Future Outlook

TMS allows the examination of functional connections between the cerebellum and the spinal cord. This method also has characteristics that are modulated by excitability changes in the cerebellar cortex, in neuronal nuclei and in extrapyramidal tracts of the brainstem. On the basis of these characteristics, it should be taken into consideration in the assessment of the functionality of the cerebellum and spinal cord in diseases that cause damage or degeneration of the cerebellum, brainstem, and spinal cord.

One clinical use of this method is the evaluation of reduced muscle tone or spinal reflex excitability, in patients with cerebellar disorders. However, since we only have reports of CSpF in two SCD cases so far, it will be necessary to gather more clinical data to examine the relationship between CSpF, abnormal muscle tone, and cerebellar disorders.

## 9. Conclusions

This review aimed to summarize recent evidence regarding the method of testing functional connectivity between the cerebellum and the spinal motoneuron pool using TMS and to present some insights for future clinical research. Recent studies have reported that cerebellar TMS can induce a motor response or facilitate the spinal reflex, and these findings suggest that the cerebellum has functional connectivity with the spinal cord. These techniques can provide insights for testing and probing the functional connectivity between the cerebellum and the spinal motoneuron pool.

## Data Availability

No personal data were used in this review.
